# Supplementary material for: Effect of inorganic material surface chemistry on structures and fracture behaviours of epoxy resin
Source: Nat Commun. 2024 Mar 8;15:1898. doi: 10.1038/s41467-024-46138-6 (PMC10923874; doi:10.1038/s41467-024-46138-6)
Supplement: Supplementary file 1 — Supplementary Information [file 41467_2024_46138_MOESM1_ESM.pdf]

Supplementary Information

# Effect of Inorganic Material Surface Chemistry on Structures and Fracture Behaviours of Epoxy Resin

Tomohiro Miyata,[1] Yohei K. Sato,[1] Yoshiaki Kawagoe,[2*] Keiichi Shirasu,[3*] Hsiao-Fang Wang,[4] Akemi Kumagai,[1] Sora Kinoshita,[5] Masashi Mizukami,[6] Kaname Yoshida,[7] Hsin-Hui Huang,[7] Tomonaga Okabe,[2] Katsumi Hagita,[8] Teruyasu Mizoguchi,[9] and Hiroshi Jinnai.[1*]

[1] Institute of Multidisciplinary Research for Advanced Materials, Tohoku University, Sendai, Miyagi 980-8577, Japan.

[2] Department of Aerospace Engineering, Graduate School of Engineering, Tohoku University, 6-6 Aramaki Aza Aoba, Aoba-ku, Sendai, Miyagi 980-8579, Japan.

[3] Department of Finemechanics, Graduate School of Engineering, Tohoku University, 6-6 Aramaki Aza Aoba, Aoba-ku, Sendai, Miyagi 980-8579, Japan.

[4] Department of Chemical and Materials Engineering, National Central University; No. 300, Zhongda Rd., Zhongli Dist., Taoyuan City 320317, Taiwan.

[5] Department of Applied Chemistry, Graduate School of Engineering, Tohoku University, 6-6 Aramaki Aza Aoba, Aoba-ku, Sendai, Miyagi 980-8579, Japan.

[6] New Industry Creation Hatchery Center, Tohoku University, Sendai, Miyagi 980-0845, Japan.

[7] Nanostructures Research Laboratory, Japan Fine Ceramics Center, Nagoya, Aichi 456-8587, Japan.

[8] Department of Applied Physics, National Defense Academy, Yokosuka, Kanagawa 239-0811, Japan.

[9] Institute of Industrial Science, the University of Tokyo, Meguro, Tokyo 153-8505, Japan.

[*] Corresponding authors:
kawagoe@tohoku.ac.jp; keiichi.shirasu.c1@tohoku.ac.jp; hiroshi.jinnai.d4@tohoku.ac.jp

**Supplementary Note 1. Thickness of the oxide layers on Si substrates**

Supplementary Table 1 presents the thicknesses of oxide layers on Si substrates, as measured by ellipsometry, after different surface treatments: piranha solution treatment (before HF), one hydrofluoric (HF) acid treatment (HF 1st), and two HF acid treatments (HF 2nd). During the HF acid surface treatment, Si substrates pre-treated with piranha solution were immersed in a 1.5 wt.% HF acid solution at room temperature for 30 min per cycle. Subsequently, the substrates were rinsed three times with pure water and dried under vacuum at room temperature. The thickness measurements were conducted within 30 min of surface treatment. The results indicate that the thickness of the oxide layer remained unchanged after the HF acid surface treatments, likely due to the dilute nature of the HF solution (1.5 wt.%). As a result, Si substrates with oxide layers of similar thicknesses but different surface chemistries were successfully fabricated. Furthermore, these thicknesses were found to be in approximate agreement with the cross-sectional ADF-STEM images of the epoxy resin–Si substrate interfaces (Figure 2).

**Supplementary Table 1.** Thicknesses of oxide layers on Si substrates measured by ellipsometry.

| Before HF | HF 1st | HF 2nd |
|---|---|---|
| $2.4 \pm 0.2$ nm | $2.8 \pm 0.3$ nm | $2.7 \pm 0.6$ nm |

**Supplementary Note 2. Contact angle of water on the OH- and H-terminated Si substrates**

Supplementary Figure 1 shows the water contact angles observed on the hydroxyl (OH)- and hydrogen (H)-terminated Si substrates, which were approximately 0° (off-scale low) and 79°, respectively. These values align with those previously reported for OH- and H-terminated Si substrates.[1,2] The OH group area density on OH-terminated Si substrates was estimated to be ~4.7 $nm^{-2}$ in a previous report.[1,3] These results indicate that the surface chemistry was successfully modified by surface treatment with water vapor plasma and HF acid. The contact angle measurements were performed within 30 min of surface treatment.

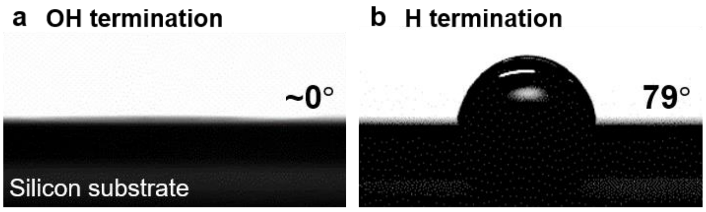


**Supplementary Figure 1.** Contact angles of water on the OH- and H-terminated Si substrates.

**Supplementary Note 3. Contact angle of DGEBA, PACM, and DGEBA–PACM mixtures on OH- and H-terminated Si substrates**

Supplementary Figure 2 illustrates the contact angles observed for bisphenol A diglycidyl ether (DGEBA), 4,4-diaminodicyclohexylmethane (PACM), and a DGEBA–PACM mixture on OH- and H-terminated Si substrates. Since these reagents are excessively viscose at room temperature, the measurements were conducted at a substrate temperature of 60 °C, which exceeds the melting temperatures of DGEBA (40–44 °C) and the glass transition temperature of PACM (30–60 °C). At this elevated temperature, DGEBA, PACM, and the DGEBA–PACM mixture remained in a low-viscosity liquid state.

From the images in Supplementary Figure 2, the contact angles of DGEBA, PACM, and the DGEBA–PACM mixture on H-terminated Si substrates were measured as 31°, 9°, and 19°, respectively. In contrast, the OH-terminated surface showed lower values of 9°, 6°, and 12°, respectively.

The contact angles ($\theta$) are expressed using Young's equation as follows:

$$\Delta\gamma_{wet} = \gamma_{SG} - \gamma_{SL} = \gamma_{LG} cos\theta$$

Here, $\gamma_{SG}$, $\gamma_{SL}$, and $\gamma_{LG}$ represent the solid–gas, solid–liquid, and liquid–gas interfacial free energies per unit area, respectively. $\Delta\gamma_{wet}$ is the difference between $\gamma_{SG}$ and $\gamma_{SL}$, reflecting the change in the interfacial free energy when a dry solid surface becomes wet with the liquid. When the surface chemistry of the solid varies, while $\gamma_{LG}$ remains constant, only $\Delta\gamma_{wet}$ differs. Therefore, smaller $\theta$ values imply a larger $\Delta\gamma_{wet}$, indicating that the liquid molecules near the solid surface are more stable.

The smaller $\theta$ values observed for each reagent on the OH-terminated substrate compared to the H-terminated substrate suggest that the reagents are more stable (with a larger $\Delta\gamma_{wet}$) on the OH-terminated surface than on the H-terminated surface. These results support the conclusion of the curing molecular dynamics (MD) simulation, indicating that the interfacial energy between the cured epoxy resin and the OH-terminated surface was more negative (more stable) than that of the H-terminated surface.

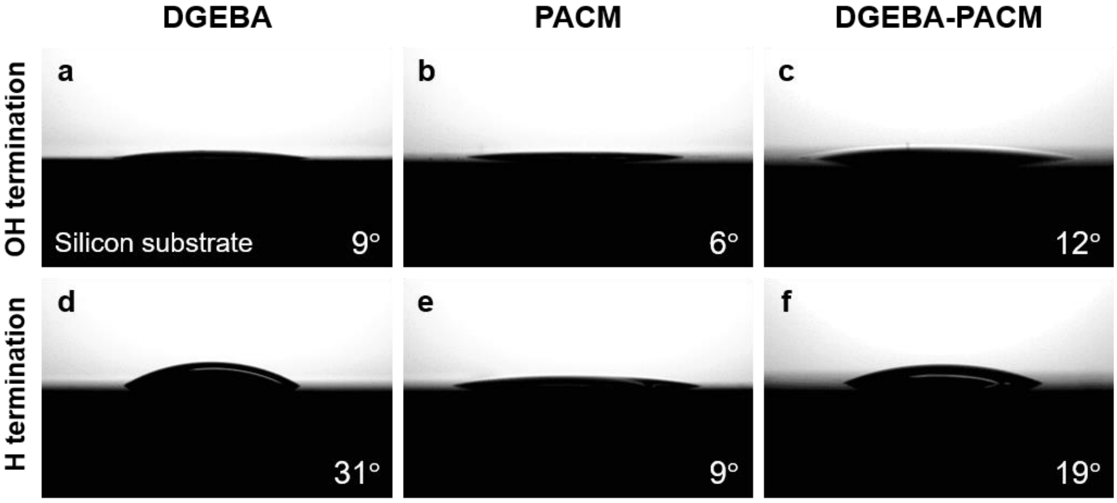


**Supplementary Figure 2.** Contact angle of DGEBA, PACM, and the DGEBA–PACM mixture of on the OH- and H-terminated Si substrates.

**Supplementary Note 4. Basics of attenuated total reflection– Fourier transform infrared spectroscopy analysis**

To explore the chemical properties near the OH- and H-terminated interfaces, attenuated total reflection–Fourier transform infrared (ATR-FTIR) spectroscopy was used to analyse the interfaces.[4] The amplitude of the electric field at a distance ($z$) away from the adhesive interface is expressed as follows:

$$E = E_0 e^{-z/d_p} \quad \text{(S1)}$$

where $E_0$ is the amplitude of the electric field at the interface ($z = 0$), $d_p$ is the penetration depth at which $E$ decays to $1/e$. The $d_p$ was calculated using the following equation:

$$d_p = \frac{\lambda}{2\pi \cdot n_1} \cdot \frac{1}{\sqrt{\sin^2\theta - (n_2/n_1)^2}} \qquad \text{(S2)}$$

where $\lambda$, $n_1$, $n_2$, and $\theta$ are the wavelength, reflective indices of the crystal (Si) and specimen (epoxy resin), and incident angle, respectively. In the present experiment, $n_1$, $n_2$, and $\theta$ were 3.4°, 1.6°, and 45°, respectively. The $d_p$ values over the wavenumber ranges of 2050–2200 cm$^{-1}$ (Supplementary Figure 3) and 2400–3600 cm$^{-1}$ (Supplementary Figure 4) were calculated to be approximately 300 and 400 nm, respectively, using Equation S2. This indicates that ATR-FTIR is sensitive to molecular vibrations (molecular structures) within several hundred nanometres of the interface. Additionally, Equation S1 shows that the amplitude of the electric field decays "exponentially" with $z$, indicating that ATR-FTIR is even more sensitive to regions close to the interface.

**Supplementary Note 5. ATR-FTIR analysis of the H-terminated Si surfaces**

Supplementary Figure 3 shows the ATR-FTIR difference spectra of the Si surfaces treated once and twice with HF acid (HF 1st and HF 2nd). These spectra were obtained by dividing the raw ATR-FTIR spectra of the 1st and HF 2nd substrates by that of the substrate before HF acid treatment. This normalization allows for the identification of changes in the surface condition resulting from the HF acid treatment.

The peaks observed in Supplementary Figure 3 (2050–2200 cm$^{-1}$) correspond to Si–H stretching vibrations, suggesting the formation of Si–H bonds on the surface of the Si substrates treated with HF acid. Furthermore, the intensities of the peaks for HF 1st (red) and HF 2nd (dotted blue) are nearly identical, indicating that the surface reached saturation in terms of Si–H bond formation after the first HF acid treatment. Subsequent HF acid treatment (HF 2nd) resulted in only a minimal increase in the number of Si–H bonds on the surface.

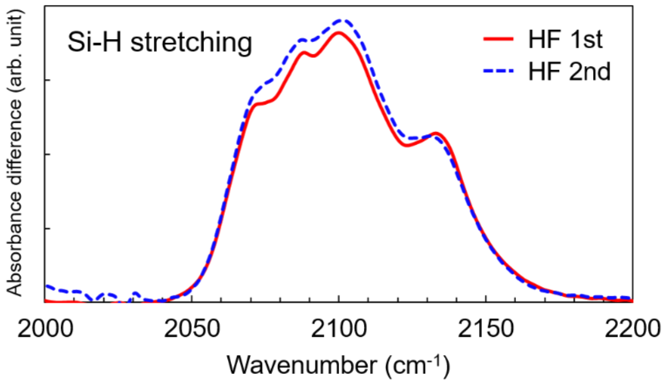


**Supplementary Figure 3.** ATR–FTIR difference spectra of the surfaces of the Si substrates treated with HF acid once (HF 1st) and twice (HF 2nd). The peaks in the range of 2050–2200 $cm^{-1}$ are attributed to Si–H bonds on the Si surfaces.

**Supplementary Note 6. ATR-FTIR composition analysis of the OH- and H-terminated interfaces**

ATR-FTIR measurements were conducted to analyse the compositional distribution of the epoxy resin near the OH- and H-terminated interfaces. The average interfacial information was extracted from the entire sample (measured area of approximately 2.5 $cm^2$). Supplementary Figure 4 depicts the ATR-FTIR spectra of the OH- and H-terminated interfaces (represented by red and blue lines, respectively). The absorbance of these ATR-FTIR spectra was adjusted by dividing it by $\lambda$ to enable a fair comparison with the transmission IR spectra. The dotted black line in Supplementary Figure 4 represents the ATR-FTIR spectrum of an interface between the epoxy resin and a silicon substrate treated with piranha solution, which also has OH termination. All spectra were normalized by matching the intensities of the peaks observed between 3020–3080 $cm^{-1}$, which are attributed to the benzene rings and epoxy groups in the DGEBA epoxy monomer.

The peaks observed at 2800–3000 $cm^{-1}$ in the spectrum correspond to the C–H stretching vibrations of the methyl ($CH_3$) and methylene ($CH_2$) groups. In particular, the peaks at 2855, 2870, 2930, and 2960 $cm^{-1}$ are attributed to the symmetric stretching vibrations of the $CH_2$ groups ($\nu_s(CH_2)$) and $CH_3$ groups ($\nu_s(CH_3)$), and the asymmetric stretching vibrations of the $CH_2$ ($\nu_{as}(CH_2)$) and $CH_3$ groups ($\nu_{as}(CH_3)$), respectively.

The OH-terminated interface spectrum exhibits higher intensities of the $CH_2$ vibrational peaks compared to those in the H-terminated interface spectrum. The intensity fluctuations and the discrepancies between the spectra of the OH- and H-terminated interfaces in the background regions (*e.g.*, 2400–2600 $cm^{-1}$ and 3120–3160 $cm^{-1}$) are more than ten times smaller than the intensity difference of the $\nu_s(\mathrm{CH_2})$ and $\nu_{as}(\mathrm{CH_2})$ peaks. Moreover, the spectrum of the interface between the epoxy resin and an OH-terminated Si substrate (treated with piranha solution) exhibited an excellent agreement with that of the OH-terminated interface. These findings strongly highlight the significance of the observed difference between the OH and H-terminated interfaces.

As the absorbances of the spectra were normalized based on the peak intensities of the DGEBA spectrum (benzene rings and epoxy groups: 2800–3000 $cm^{-1}$), the peak intensities of the $CH_3$ groups, which are exclusively present in DGEBA, were assumed to be nominally the same for all spectra. However, in the spectrum of the H-terminated interface, the apparent decrease in the peak intensity of $\nu_s(\mathrm{CH_3})$ is attributed to the smaller background of the $\nu_{as}(\mathrm{CH_2})$ peak.

The larger peak intensities of $CH_2$ vibrations observed at the OH-terminated interface compared to the H-terminated interface indicate a higher fraction of PACM relative to DGEBA near the OH-terminated interface. It is important to note that the depth resolution of ATR-FTIR remains limited in determining the precise compositional distribution near the interfaces, as the information is averaged in the depth direction according to Equation S1. However, this result aligns with the findings from STEM-EELS analysis (C-K and N-K edges), which also supports the notion of PACM condensation as a common characteristic throughout the entire OH-terminated interface.

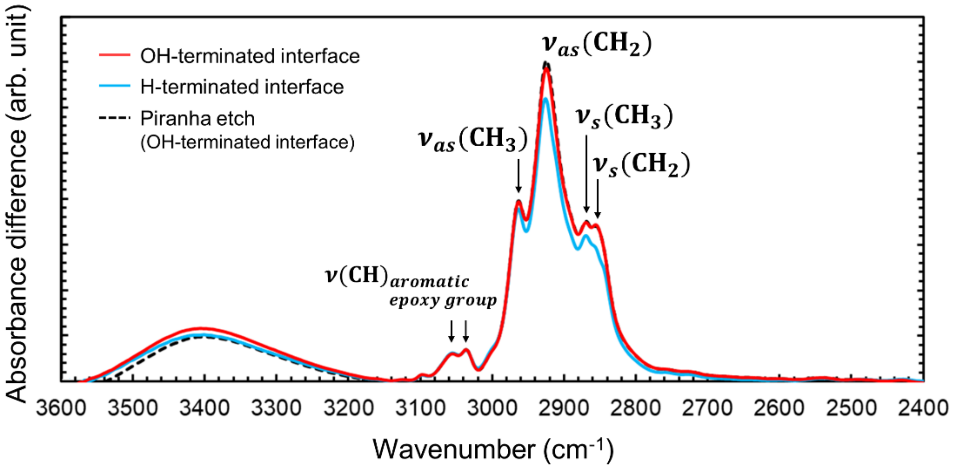


**Supplementary Figure 4.** ATR-FTIR spectra of the OH- and H-terminated interfaces, along with that of an interface between the epoxy resin and a Si substrate treated with piranha solution, which also has an OH-terminated interface. The absorbance of the ATR-FTIR spectra was adjusted to enable comparison with the transmission FTIR spectrum. The absorbances of all spectra were normalized based on the intensity of the peaks corresponding to the benzene rings and epoxy groups (3020–3080 $cm^{-1}$) present in DGEBA.

**Supplementary Note 7. Cross-linking mechanisms of the DGEBA–PACM epoxy resin**

Supplementary Figure 5 illustrates the chemical reaction pathways of DGEBA and PACM, which involve two stages: (1) the reaction between the primary amine and epoxy groups, and (2) the reaction between a secondary amine and epoxy groups. The schematic and curing MD simulations conducted in this study do not consider the reaction between an amine and a hydroxyl group in the reacted DGEBA (etherification reaction).

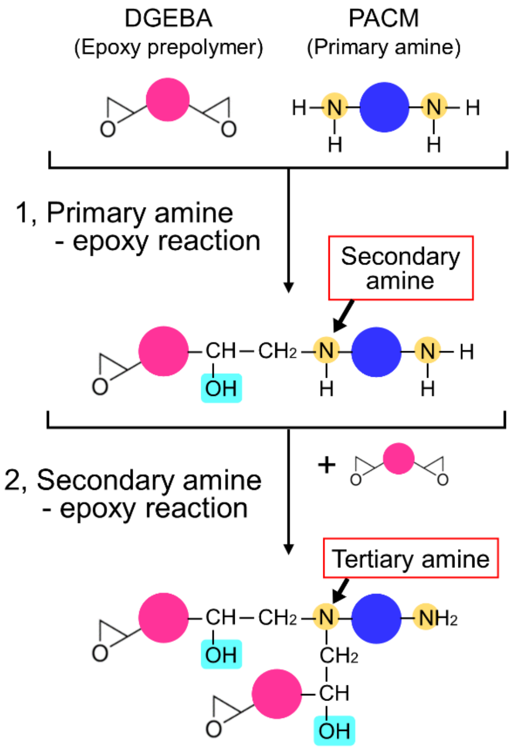


**Supplementary Figure 5.** Curing mechanism of the chemical reactions of DGEBA and PACM.

**Supplementary Note 8. Si-$L_{2,3}$ edge ELNES of the Si atoms at the $SiO_2$ (111) surfaces with different chemical bonds**

Supplementary Figure 6 presents the structural models used for the Si-$L_{2,3}$ edge ELNES simulation. Supplementary Figure 6(a) depicts the original $SiO_2$ (111) surface structure terminated with Si–O–Si bonds. Supplementary Figure 6(b) shows stable H-terminated surfaces, where the original surface structure was modified by incorporating four H atoms per unit cell. Supplementary Figure 6(c) shows model structures of the variously modified $SiO_2$ (111) surfaces, where a surface Si atom forms a bond with a H atom, OH group, O-phenyl group, as well as O–C(–O)–R, N–R, and C–R groups.

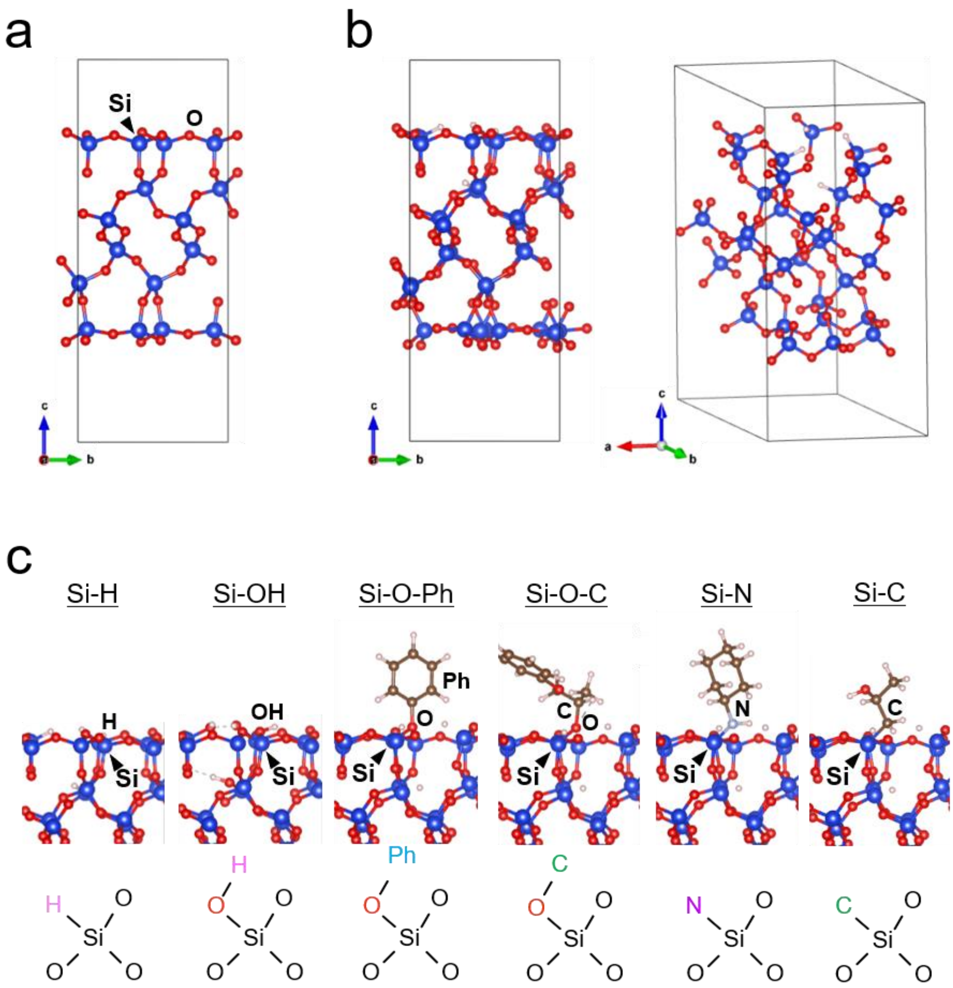


**Supplementary Figure 6.** Structure models for the Si-$L_{2,3}$ edge electron energy-loss near-edge structure (ELNES) simulation.

Supplementary Figure 7 depicts the simulated ELNES spectra of the Si-$L_{2,3}$ edges for the structural models illustrated in Supplementary Figure 6. The peaks located at 107.5, 109, and 115 eV (marked by green triangles in Supplementary Figure 7) exhibit characteristic features of $SiO_2$. Notably, the spectra of the Si–H and Si–C surface models display distinct profiles, featuring enhanced peak intensities at lower energy (~106 eV), while the peak intensities around 109 and 115 eV are suppressed compared to those of $SiO_2$. In contrast, the spectra for Si–OH, Si–O–Ph, Si–O–C, and Si–N demonstrate similarities to the spectrum of $SiO_2$, although each spectrum is slightly shifted towards higher energy by approximately 0.5 eV.

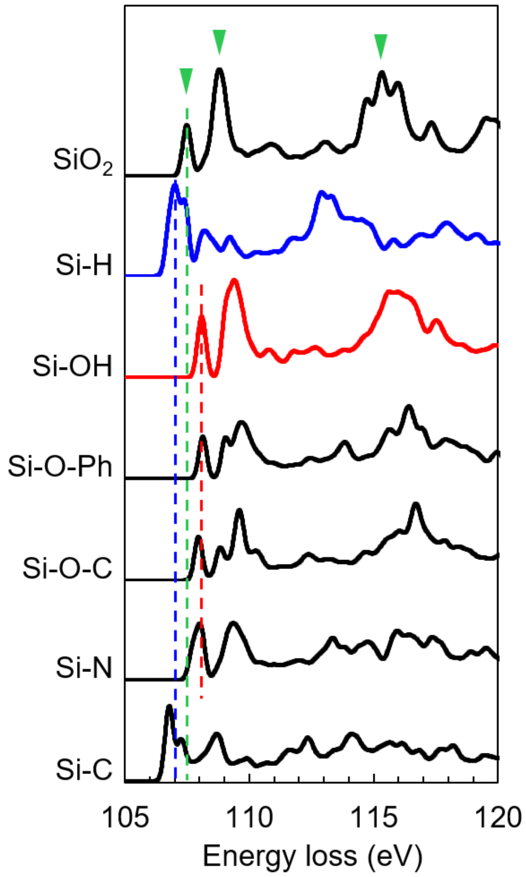


**Supplementary Figure 7.** Simulated Si-L2,3 edge ELNES spectra corresponding to the Si atoms indicated with black triangles in Supplementary Figure 6(c).

**Supplementary Note 9. N-K edge spectra near the OH- and H-terminated interfaces**

Supplementary Figure 8 shows the N-K edge spectra, measured at 2 nm intervals, spanning from the OH- and H-terminated interfaces towards the epoxy resin. The profiles indicating the number of nitrogen atoms (representing PACM molecules) close to the interfaces were determined by integrating the intensity of the N-K edge spectrum within the energy range of 400–420 eV at each distance from the epoxy resin–Si substrate interface. Additionally, to eliminate the influence of specimen thickness, the integrated intensities of the N-K edge spectra were normalized using the C-K edge intensities integrated within the energy range of 280–300 eV at corresponding positions. The resulting normalized nitrogen profiles (N-K/C-K) for the OH- and H-terminated Si substrates are presented in Supplementary Figure 9. The error bars were defined

as the deviation from the average values in the regions within 5 – 15 nm from the interfaces, assuming that the stoichiometric ratio would be uniform in the region.

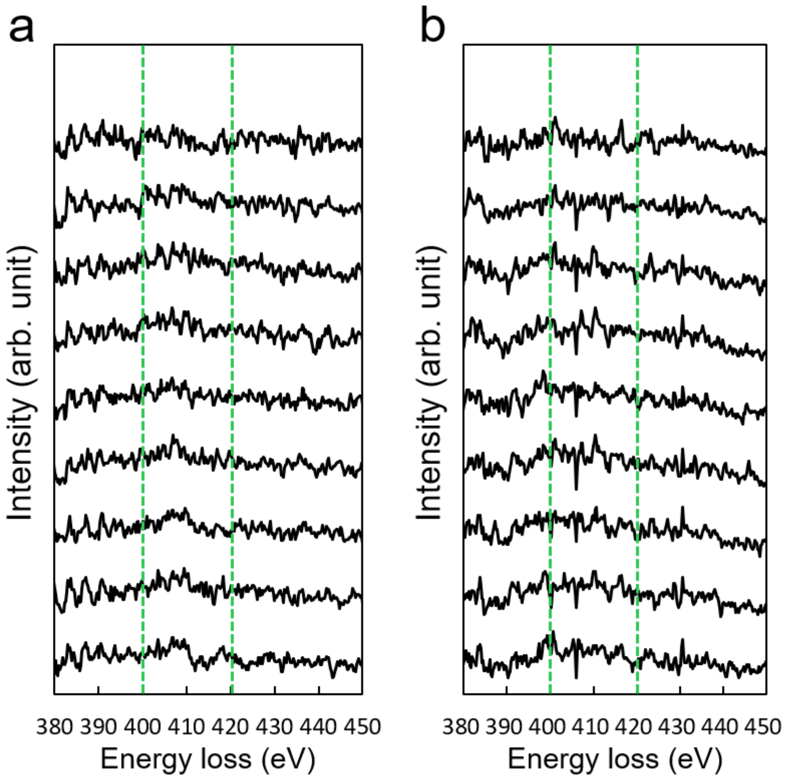


**Supplementary Figure 8.** N-K edge spectra near the interfaces between the epoxy resin and (a) OH- and (b) H-terminated Si substrates.

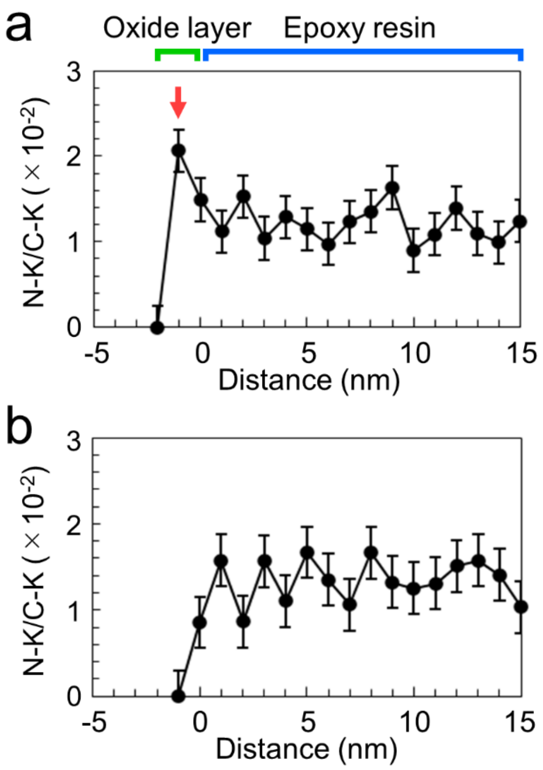


**Supplementary Figure 9.** Profiles of the integrated intensity of the N-K edge spectrum divided by that of the corresponding C-K edge spectrum (N-K/C-K) measured at different distances from the adhesive interfaces. Profiles of the (a) OH-terminated and (b) H-terminated interfaces. The error bars were defined as the deviation from the average values in the regions within 5 – 15 nm from the interfaces, assuming that the stoichiometric ratio would be uniform in the region.

**Supplementary Note 10. Estimations of the stoichiometric ratios from STEM-EELS spectra**

The stoichiometric ratios of PACM to DGEBA were evaluated from EELS data by using the following two approaches.

(i) N-K peak vs. C-K peak (N-K/C-K)

The integrated intensities of the N-K and C-K edge spectra ($I_{N-K}$ and $I_{C-K}$) were converted to the ratio the density of the N atoms in PACM ($N_N$) to that of the C atoms in both DGEBA and PACM ($N_C$) using a *k* factor as follows[5].

$$\frac{N_N}{N_C} = k \cdot \frac{I_{N-K}(\beta,\Delta_{N-K})}{I_{C-K}(\beta,\Delta_{C-K})} \quad \text{(S3)}$$

where $\beta$ is the collection angle of the EELS spectrometer, and $\Delta$ is the energy range used to integrate the spectral intensity. In this study, $\Delta_{C-K}$ and $\Delta_{N-K}$ were set to 280–310 and 390–420 eV for the C-K and N-K edge spectra, respectively. The $k$ factor (a constant value independent of the stoichiometric ratio) was calculated by measuring the intensity ratio ($I_{N-K}/I_{C-K}$) from the epoxy resin with a stoichiometric ratio of 1.0, where $N_N/N_C$ should be 2/55. Once the $k$ factor was determined, the stoichiometric ratio at each position in the epoxy resin near the interfaces was estimated by dividing the right term in Equation S3 ($k \cdot I_{N-K}/I_{C-K}$) by 2/55. Here, we assumed that the stoichiometric ratio of the epoxy resin in the regions 10–15 nm away from the interface was 1.0 for both OH- and H-terminated specimens.

(ii) C-K π* peak vs. C-K σ* peak (C-K π*/σ*)

The stoichiometric ratio of PACM to DGEBA was evaluated from the intensity ratio of a C-K π* peak to σ* peak. The π* peak intensities of C-K spectra reflect the amount of the C atoms in the benzene rings of the DGEBA molecules. The intensities of C-K σ* peaks represent the C atoms not included in the benzene rings. Spectral fitting with three Gaussian functions was performed for the C-K edge spectra as shown in Supplementary Figure 10. Whereas the first peak ($P_1$) corresponds to the π* peak and the second ($P_2$) and third ($P_3$) peaks represent the other peaks. The ratio of the $P_1$ intensity to the $P_2$+ $P_3$ intensities is described as follows.

$$\frac{N_\pi}{N_{all}} = k \cdot \frac{I_{P_1}}{I_{P_2}+I_{P_3}} \tag{S4}$$

Here, $N_\pi$ is the number of C atoms in the benzene rings in DGEBA, and $N_{all}$ is the total number of C atoms consisting of DGEBA and PACM in the epoxy resin. The $k$ factor was determined in the same way as described in Equation S3. The ratio of $N_\pi/N_{all}$ should be 24/55 for a stoichiometric ratio of 1.0. Therefore, the stoichiometric ratio at each position in the epoxy resin near the interfaces was estimated by dividing the right term in Equation S4 ($k \cdot I_{P_1}/(I_{P_2} + I_{P_3})$) by 24/55.

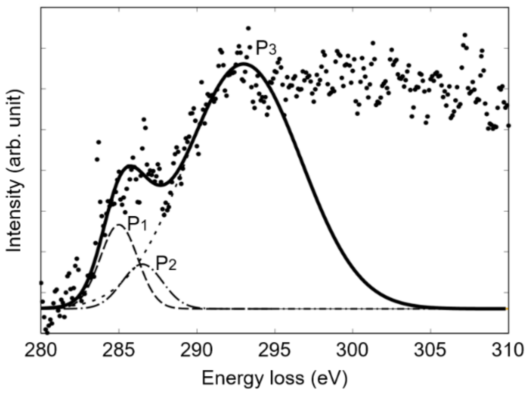


**Supplementary Figure 10.** Decomposition of a C-K absorption spectrum. Three gaussian functions were applied for the spectral fitting.

**Supplementary Note 11. Stoichiometric ratios calculated from the cured epoxy resin structures**

The simulations did not reproduce the significant differences in the stoichiometric ratios near the interfaces observed by STEM-EELS because the simulation conditions did not match those of the experimental systems (e.g. small system size and short calculation time). Thus, the average stoichiometric ratios of the uncured epoxy resins within the OH- and H-terminated systems were adjusted to 1.4 and 0.8, respectively, to produce the interfacial compositions relevant to those measured by STEM-EELS. Then, the curing MD simulations were performed on these systems. We note that the simulations did not consider chemical reactions between the epoxy resin molecules and the substrates.

Supplementary Figure 11 illustrates the 1-nm-averaged stoichiometric ratios against the distance from the OH- and H-terminated interfaces. The red and blue squares indicate the stoichiometric ratios of the epoxy resins for the systems with the OH- and H-terminated interfaces, respectively. The stoichiometric ratios were calculated from the ratios of the number of the C atoms in the benzene rings of DGEBA to that of the C atoms not in the benzene rings. This approach to calculating the stoichiometric ratios corresponds to that used for the experimental C-K π*/σ* analysis.

The stoichiometric ratios within 1 nm from the OH- and H-terminated interfaces (grey region in Figure 7(b)) are 1.5 and 1.0, respectively, which are similar to the stoichiometric ratios near the OH- and H-terminated interfaces as measured by STEM-EELS. We examined the molecular structures within the 1-nm region from the interfaces (grey shaded region) that represents the experimental results.

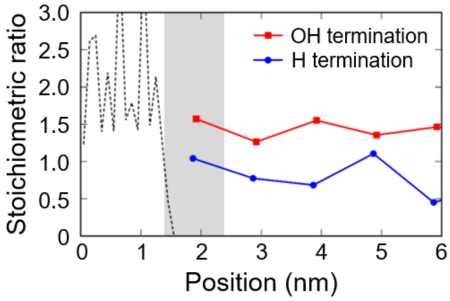


**Supplementary Figure 11.** Profiles of the stoichiometric ratios of the simulated epoxy resin structures for the OH- and H-terminated interfaces. The black dashed line represents the number densities of Si and O atoms in the substrates, while the grey shaded region is the 1-nm region corresponding to the experimental results.

**Supplementary Note 12. Lap-shear and tensile adhesive strength tests**

Lap-shear and adhesive strength tests were used to measure the adhesive strengths between the epoxy resin and silicon substrates. Supplementary Figure 12 shows photographs and schematics of the lap-shear test setup and procedure. The adhesive strengths of the OH- and H-terminated interfaces were measured to be 34.8 ± 1.2 and 30.8 ± 1.8 MPa, respectively. Due to the brittleness of the silicon substrates, the success rate of the lap shear test is very low. The pictures of representative successful and failed pieces are shown in Supplementary Figure 12. The successful piece exhibits a mirror-like silicon surface exposed by the interfacial delamination between the epoxy resin and silicon substrate. The failed piece shows a broken silicon substrate.

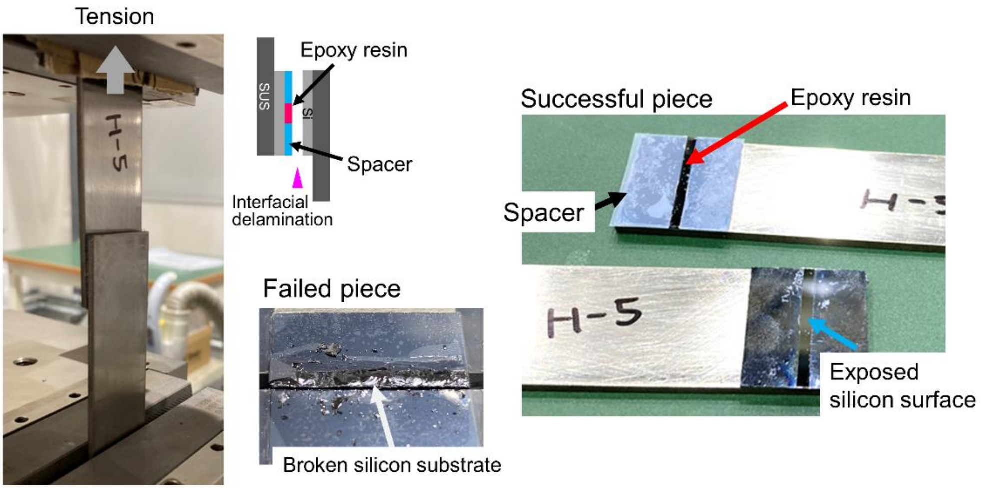


**Supplementary Figure 12.** Schematics of the lap-shear test and successful and failed pieces.

Supplementary Figure 13 shows schematics of the tensile adhesive strength test setup. In the test, two OH- and H-terminated Si substrates, each measuring 25 mm in width and 25 mm in length, were bonded with the DGEBA–PACM epoxy resin. A single-sided release film with a rectangular hole (25 mm width and 5 mm length) filled with a mixture of DGEBA and PACM was sandwiched between the two Si substrates. The assembly was then heated at 100 °C for 90 min to cure the epoxy resin. The region of the epoxy resin measured 25 mm in width, 5 mm in length, and 0.19 mm in thickness.

Furthermore, SUS304 blocks, measuring 25 mm in width, 25 mm in length, and 12 mm in thickness, were bonded to both sides of the test pieces using a room-temperature curing adhesive. The test pieces were mounted on a universal testing machine (Instron 5567, Instron Corporation, USA) with a 20 kN static load cell. The tensile adhesive strength tests were conducted at a tensile speed of 0.02 mm $min^{-1}$. The adhesive strength was calculated by dividing the load-at-break by the adhesion area of 25 mm × 5 mm.

As a result, we obtained the adhesive strengths of 11.4 ± 1.6 and 10.4 ± 3.9 MPa for the OH- and H-terminated interfaces, respectively. Although the errors for both values are large, the mean values (11.4 and 10.4 MPa) imply a similar strength relationship as those measured by the lap shear test.

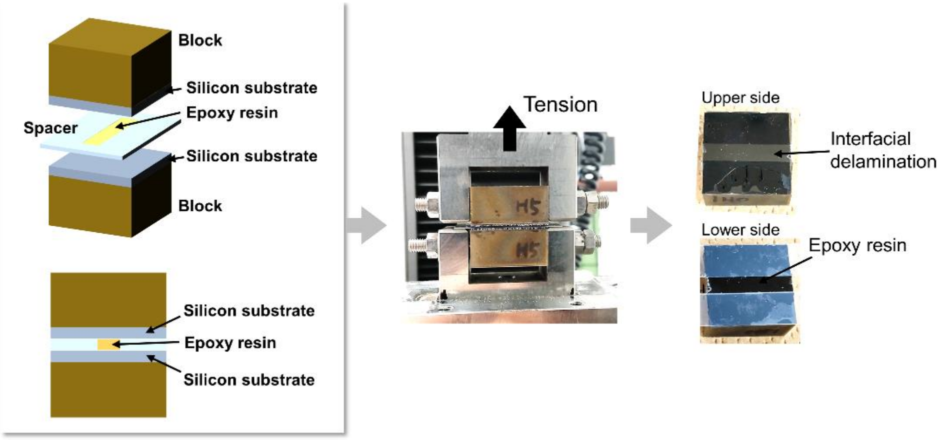


**Supplementary Figure 13.** Schematics and photographs of the tensile adhesive strength test setup.

**Supplementary Note 13. Stress-field analysis around a crack tip at the epoxy resin-silicon substrate interfaces**

A finite element model (FEM) of the interfacial specimens used for the TEM observation of the fracture surfaces was created in the explicit finite element code LS-Dyna. As shown in Supplementary Figure 14, the shapes and dimensions of the model were set to replicate the specimen shown in Figure 8(b). The materials properties of the Si substrate and epoxy resin were defined by isotropic elastic and isotropic elastoplastic models, respectively. The material models of *MAT_001 and *MAT_012 were used for the simulation in the LS-Dyna software. The equivalent stress and work hardening rate, which were input into *MAT_012, were determined based on the stress–strain curves obtained from uniaxial tensile tests and FEM analysis of the epoxy resin. The model was composed of the epoxy resin and silicon parts with C3D8 elements across the 300 nm thickness. The model had 94,772 nodes and 70,152 elements. The model was divided into three sections of 100 nm in the thickness direction. The widths and lengths of the divisions were set in the range of 10–200 nm and 10–500 nm, respectively. The elements at the edges of the epoxy resin–silicon interface were divided into 10 nm × 10 nm × 100 nm volumes.

Assuming that crack formation occurs at the ends of the interface due to the stress concentration involved in the Poisson shrinkage, pre-cracks were introduced in the elements at both ends of the interface. No

fracture was considered for the other parts of the interface. To reproduce the experimental setup (Figure 8b), while the displacement of the hatched area on the silicon substrate (representing the nodes on the silicon surface) in Supplementary Figure 14 was constrained, the nodes in the hatched area on the epoxy resin were subjected to displacement in the *y* direction. The macroscopic tensile adhesive-strength tests of the epoxy resin–Si substrate interfaces (where tensile loads were applied perpendicularly to the interfaces) exhibited delamination at ~10 MPa. Therefore, the stress distribution at the pre-crack tip was analysed with a simulated tensile stress of 9.8 MPa, assuming that the delamination occurred at the similar stress in the interfacial specimens used for TEM observations.

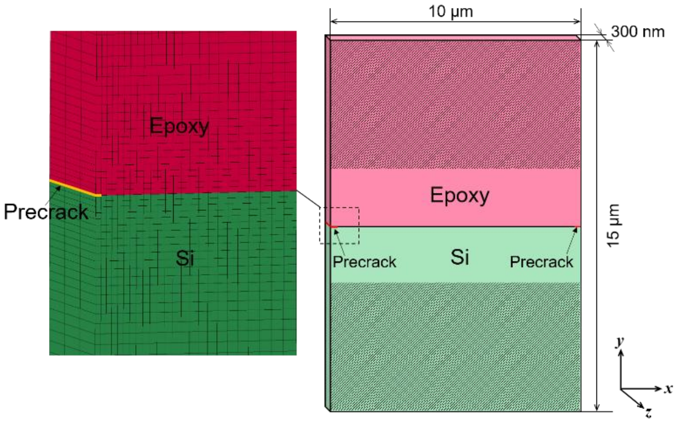


**Supplementary Figure 14.** Schematic illustration of the finite element model and mesh at the epoxy resin–silicon substrate interface.

Figure 15 shows the stress–strain curve obtained from the FEM analysis. As mentioned above, this analysis did not model the fracture or interfacial delamination between the epoxy resin and silicon substrate. The tensile loading was applied until the epoxy resin plastically deformed. The experimentally obtained adhesive strength (~10 MPa) is in the elastic-deformation region where the stress is linearly increasing. Figure 16 shows the stress distribution near the interface at ~10 MPa and variation in the tensile stress $\sigma_y$ of the epoxy resin elements close to the interface, which is plotted as a double logarithmic graph against

the distance $r$ along the interface from the crack tip. Because we assumed that the cracks were generated at the epoxy resin–silicon interface, although the relationship between $\sigma_y$ and $r$ did not show the singularity of $1/\sqrt{r}$, there was no process zone and HRR singular field. Therefore, the crack starts to propagate once the stress intensity factor at the crack tip reaches the fracture toughness value of the interface. In other words, the area outside the pre-crack is the region where the crack propagates rapidly during the final fracture stage. In this case, the crack propagates along with the fracture occurring at regions with weak bonds located in the direction of crack propagation. If the adhesive strength of the epoxy resin–silicon interface is less than the cohesive strength of the epoxy resin, the crack propagates along the interface, and vice versa.

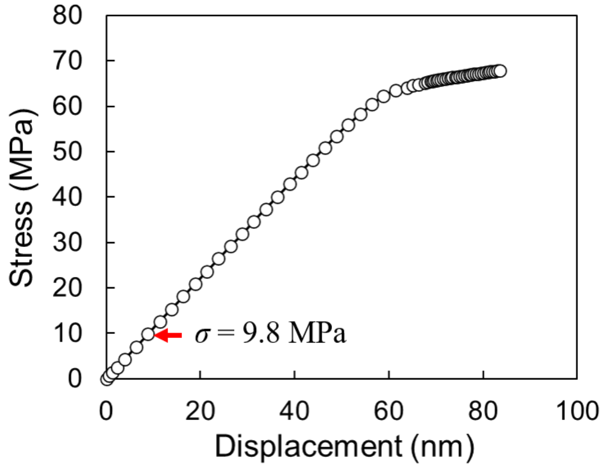


**Supplementary Figure 15.** Simulated stress–strain curve of the epoxy resin–Si interfacial model.

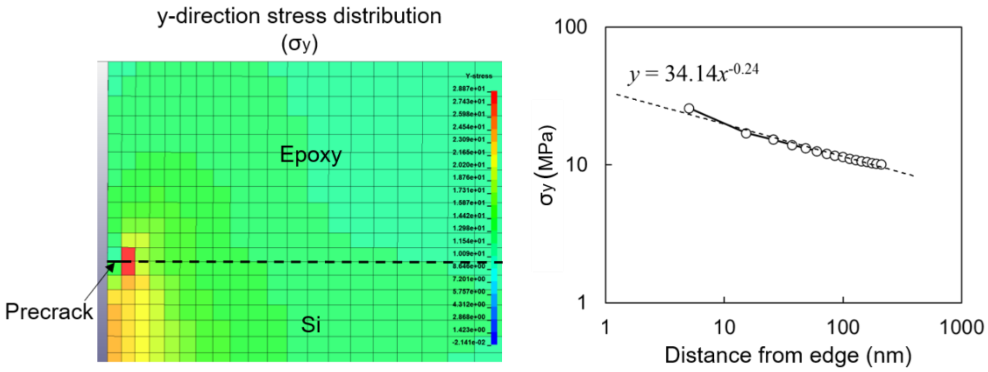

**Supplementary Figure 16.** (a) Distribution of tensile stress ($\sigma_y$) around the interfacial crack tip and (b) $\sigma_y$ distribution along the interface between the epoxy resin and Si substrate.

## Supplementary references


(1) Emami, F. S.; Puddu, V.; Berry, R. J.; Varshney, V.; Patwardhan, S. V.; Perry, C. C.; Heinz, H. Force Field and a Surface Model Database for Silica to Simulate Interfacial Properties in Atomic Resolution. *Chem. Mater.* **2014**, *26* (8), 2647–2658. https://doi.org/10.1021/cm500365c.

(2) Adachi, S.; Arai, T.; Kobayashi, K. Chemical Treatment Effect of Si(111) Surfaces in F-Based Aqueous Solutions. *J. Appl. Phys.* **1996**, *80* (9), 5422–5426. https://doi.org/10.1063/1.362729.

(3) Ewing, C. S.; Bhavsar, S.; Veser, G.; McCarthy, J. J.; Johnson, J. K. Accurate Amorphous Silica Surface Models from First-Principles Thermodynamics of Surface Dehydroxylation. *Langmuir* **2014**, *30* (18), 5133–5141. https://doi.org/10.1021/la500422p.

(4) Mizukami, M.; Nakagawa, Y.; Kurihara, K. Surface Induced Hydrogen-Bonded Macrocluster Formation of Methanol on Silica Surfaces. *J. Am. Chem. Soc.* **2002**, *124* (43), 12889–12897. https://doi.org/10.1021/la0512190.

(5) Egerton, R. F. *Electron Energy-Loss Spectroscopy in the Electron Microscope, 3rd Edition*; Springer New York: N.Y., 2011.
