# Supplementary material for: Effect of inorganic material surface chemistry on structures and fracture behaviours of epoxy resin
Source: Nat Commun. 2024 Mar 8;15:1898. doi: 10.1038/s41467-024-46138-6 (PMC10923874; doi:10.1038/s41467-024-46138-6)
Supplement: Supplementary file 3 — Description of Additional Supplementary Files [file 41467_2024_46138_MOESM3_ESM.pdf]

Description of Additional Supplementary Files

File Name: Supplementary Data 1
Description: Raw data of Figure 6. Atomic coordinates of the cured epoxy resins with various stoichiometric ratios in bulk systems.

File Name: Supplementary Data 2
Description: Raw data of Figure 7. Atomic coordinates of the cured epoxy resins with various stoichiometric ratios in the interface systems.
